# Delayed primary closure versus subcutaneous suction drain with primary closure following contaminated laparotomy wound

**DOI:** 10.6026/973206300222640

**Published:** 2026-04-30

**Authors:** Arkaprovo Roy, Prodipta Mondal, Rajarshi Kumar

**Affiliations:** 1 Department of Surgery, North Bengal Medical College and Hospital, Siliguri, West Bengal, India; 2 Department of General Surgery, Medical College & Hospital, Kolkata, West Bengal, India; 3 Department of Pediatric Surgery, North Bengal Medical College and Hospital, Siliguri, West Bengal, India

**Keywords:** Delayed primary closure (DPC), subcutaneous suction drain, contaminated laparotomy, surgical site infections (SSIs), emergency abdominal surgery

## Abstract

Surgical site infections (SSIs) remain a major cause of morbidity and prolonged hospitalization following emergency contaminated laparotomies and the optimal method of skin closure in such wounds is still debated. Therefore, it is of interest to evaluate delayed primary closure (DPC) versus primary closure with subcutaneous suction drain (PC-D) in 120 adults undergoing emergency contaminated laparotomies at a tertiary care centre between January 2023 and June 2025. SSIs occurred in 13.3% of patients in the DPC group and 23.3% in the PC-D group, a difference that did not reach statistical significance, while hospital stay was significantly longer in the DPC group. Readmission rates and early SSI rates were comparable between groups and diabetes, obesity and hypoalbuminemia were identified as independent predictors of SSI. Although DPC showed a trend toward lower SSI rates, it was associated with longer hospitalization, suggesting that wound closure strategies in contaminated laparotomies should be individualized, particularly in high-risk patients. In high-risk patients (e.g., diabetics), DPC may be superior. Thus, we recommend for tailored approaches in contaminated wounds, with larger trials needed for definitive guidelines.

## Background:

Surgical site infections (SSIs) are also one of the most common forms of nosocomial infections with more than 2 million surgical patients in the United States alone, affected every year [1]; globally, a prospective cohort study across 66 countries found SSI rates of 11.8% for clean-contaminated procedures, with low- and middle-income countries bearing a disproportionate burden [2]. Not only do these infections prolong hospital stay and increase expenses but they have doubled mortality and fivefold readmission rates [3]. According to the Centers for Disease Control and Prevention (CDC), SSIs are infections that appear within 30 days after the surgery (or one year in case of implants) and appear at the incision site with pus, tenderness or systemic symptoms [4]. The frequency of SSI in contaminated laparotomy wounds can reach its highest levels in Class III (gross spillage of gastrointestinal tract or acute non-purulent inflammation) and Class IV (purulent inflammation or perforated viscera) [5]. The etiology of SSIs is multifactorial and includes elements related to patients (such as advanced age, malnutrition, hypovolemia, obesity, diabetes, smoking and immunosuppression), as well as procedural factors (long operative time and the level of contamination) [6]. Emergency laparotomies (including perforated viscera and trauma) do not allow the optimization of preoperative condition, which increases risks to more than 40% [7]. A multinational prospective cohort study further confirmed that emergency abdominal surgery is independently associated with significantly higher postoperative morbidity and mortality compared to elective procedures, underscoring the critical need for optimized wound management protocols in this setting. Such pathogens as *Staphylococcus aureus* (including MRSA), *Enterococcus spp*. and *Escherichia coli*, which thrive in violated sterile environments, are common [8]. According to CDC guidelines, the antimicrobial prophylaxis, antiseptic skin preparation using chlorhexidine-alcohol, normothermia (>36 C) and glycemic control are included in preventive strategies included since 1999 [4]. The WHO updated its Global Guidelines for SSI Prevention in 2021, reinforcing these evidence-based measures and adding recommendations on wound irrigation, antimicrobial sutures and perioperative oxygenation, providing a global framework applicable to resource-variable settings such as India. Other measures, such as doubling of gloves, sterilization of equipment and reduction of the traffic in the operating room, also reduce contamination [9]. Registered SSIs are treated using wound debridement, moist dressing to promote granulation and epithelialization and deep or systemic involvement with systemic antibiotics [10]. Hyperbaric oxygen therapy (75 percent success in non-healing wounds) and vacuum-assisted closure (VAC) are examples of advanced adjuncts used in healing, which help in reducing edema and improving perfusion [11]. Nonetheless, in infected wounds, the choice of closure method has a key role to play. The 2023 World Society of Emergency Surgery (WSES) ECLAPTE guidelines further affirmed that there is currently no consensus on the optimal skin closure method in emergency laparotomy and called for risk-stratified, individualized decision-making informed by contamination class and patient risk factors [17]. Delayed primary closure (DPC) was introduced by H.H. Hepburn in 1919 in war victims; it involves open dressing, 3-5 days to reduce the number of bacteria and enhance oxygenation in the wound, followed by suture [12]. A recent 2024 meta-analysis of RCTs confirmed that DPC significantly reduces SSI rates following surgery for gastrointestinal perforation compared to primary closure, supporting the preferential use of DPC in this high-risk subgroup [18]. Although DPC may be effective to decrease SSIs in certain studies [13], it also raises the cost of care, dressing, length of stay and pain [14]. On the other hand, primary closure with subcutaneous suction drain (PC-D) closes dead space, empties seroma and has been promoted in high-risk groups such as the obese, where it has been reported that it results in similar or less SSIs in abdominal surgeries [15]. However, there is also contradictory evidence: according to some meta-analyses, drains do not have any impact because they can act as the means of infection [16]. The prevalence of SSI in India remains unestimated, with little local evidence on DPC and PC-D and emergency laparotomies strain resources, which results in limited studies on the topic to date. A recent prospective comparative study from an Indian tertiary care centre similarly reported that DPC significantly reduced SSI rates compared to primary closure in Class III/IV laparotomy wounds, underscoring the relevance of this question in resource-limited settings [19]. This discrepancy highlights the necessity of context-related information to reduce morbidity and costs. Our research is concerned with the estimation of the SSI rates at a tertiary care centre and comparing the DPC and PC-D effectiveness in contaminated emergency laparotomy. Research questions are the following: 1) SSI frequency; 2) comparative SSI decrease; 3) the influence of comorbidities, BMI, previous surgeries, formation of a stoma and blood parameters.

## Hypothesis:

Both methods are used in the management of contaminated wounds, but one of them can better prevent SSIs, which directs the best practices in such environments. This novel trial will help to form guidelines to potentially reduce stays and psychological burdens of SSIs.

## Materials and Methods:

This prospective randomized controlled trial was conducted at the Emergency Department of a tertiary care centre from January 2023 to June 2025, following approval from the Institutional Ethics Committee (IEC/MCK/2022/045) and registration with the Clinical Trials Registry of India (CTRI/2023/01/048765). Informed written consent was obtained from all participants or their guardians.

## Study population and sample size:

Adult patients (18-70 years) undergoing emergency open laparotomy for contaminated wounds (CDC Class III: acute non-purulent inflammation, gross gastrointestinal spillage, or traumatic wounds 12-24 hours old) were included. Exclusion criteria encompassed clean/ clean-contaminated or dirty/infected wounds, pregnancy, immunosuppression (e.g., HIV, chemotherapy), coagulopathy and refusal of consent. Sample size was calculated using OpenEpi software, assuming SSI rates of 20% in PC and 10% in DPC based on prior literature (power 80%, alpha 0.05, 1:1 allocation), yielding 57 patients per group; we recruited 60 per group (n=120) to account for attrition.

## Randomization and interventions:

Patients were randomized using computer-generated blocks (block size 4) into Group A (DPC) and Group B (PC with subcutaneous suction drain). Allocation was concealed in opaque envelopes. All surgeries followed standard protocols: preoperative antibiotics (ceftriaxone 1g IV + metronidazole 500mg IV), chlorhexidine skin preparation and midline incisions. In Group A, skin was left open, dressed with saline- soaked gauze changed daily for 3-5 days, then sutured if no infection signs. In Group B, skin was closed primarily with nylon sutures and a closed subcutaneous suction drain (Romovac, size 14Fr) was placed, removed when output <30ml/day (typically 3-5 days). Postoperative care included antibiotics for 5 days, daily wound inspections and analgesia.

## Outcome measures:

Primary outcome was SSI incidence within 30 days, diagnosed clinically (erythema, pus, and tenderness) and graded via Southampton Wound Scoring System (Grade 0: normal healing; Grade IV: deep infection). Secondary outcomes included hospital stay duration, readmission rate, early SSI (within 3 days) and effects of covariates (comorbidities, BMI, hemoglobin, albumin, stoma formation, previous surgery). Data were collected via structured proforma and follow-up calls/clinics.

## Statistical analysis:

Data were analyzed using SPSS version 26.0 (IBM Corp., Armonk, NY). Normality was assessed with Shapiro-Wilk test. Categorical variables (e.g., SSI rates) were compared using chi-square or Fisher's exact test; continuous variables (e.g., hospital stay) with independent t-test or Mann-Whitney U test. Multivariate logistic regression identified SSI predictors, adjusting for confounders (age, diabetes, BMI). Kaplan-Meier curves estimated time-to-SSI, compared via log-rank test. P<0.05 was significant. Intention-to-treat analysis was employed, with missing data handled by multiple imputations.

## Results:

A total of 120 patients were randomized (60 per group); baseline characteristics were comparable (Table 1 - see PDF). Mean age was 44.8 ± 11.9 years in Group A and 45.6 ± 12.7 years in Group B; males comprised 63.3% and 66.7%, respectively. Comorbidities included diabetes (23.3% vs 26.7%) and obesity (BMI>30: 18.3% vs 21.7%). Baseline hemoglobin was 11.2 ± 1.8 g/dL in Group A and 11.4 ± 1.6 g/dL in Group B. The primary outcome, SSI within 30 days, occurred in 22 patients (18.3%). Group A (DPC) had 8 SSIs (13.3%), versus 14 in Group B (23.3%) (Chi-square=2.18, p=0.14; Table 2 - see PDF). Most SSIs were superficial (Grade II-III: 72.7%). Early SSI (within 3 days) was 3.3% in Group A and 5% in Group B (p=0.68). Mean hospital stay was significantly longer in Group A (12.4 ± 3.2 days) than Group B (9.8 ± 2.7 days) (t=4.72, p<0.001; Figure 1 - see PDF). Readmission rates: 5% (3/60) in Group A (all for wound dehiscence), 8.3% (5/60) in Group B (p=0.49). Multivariate logistic regression revealed diabetes (OR=2.8, 95% CI 1.2-6.5, p=0.02), BMI>30 (OR=3.1, 95% CI 1.3-7.4, p=0.01) and low albumin (<3.5 g/dL) (OR=2.4, 95% CI 1.1-5.3, p=0.03) as independent SSI predictors. Stoma formation increased risk (OR=2.6, p=0.04), while previous surgery did not (p=0.21). No significant group differences in blood parameters (hemoglobin, leukocytes) post-surgery (p>0.05). Kaplan-Meier analysis showed median time-to-SSI of 8 days in Group A versus 6 days in Group B (log-rank p=0.12). Subgroup analysis: In diabetic patients, DPC reduced SSI (10% versus 30%, p=0.04). There were no statistically significant differences between the two groups with respect to mean age and baseline hemoglobin levels (p > 0.05; Figure 2 - see PDF). In contrast, the duration of hospital stay differed significantly between the groups, favoring primary closure with drain (Figure 2 - see PDF).

## Discussion:

We found that there was a nonsignificant tendency of reduced SSIs with DPC (13.3%) compared to PC-D (23.3%; p=0.14) in contaminated emergency laparotomies and extended stays with DPC (p<0.001). The SSI rate is 18.3% in total and is consistent with literature on Class III wounds with a range of 15-40% [1, 4]. This reinforces the mechanism of bacterial reduction and improved oxygenation that is evident during open dressing with DPC, indicated by Siribumrungwong *et al.* RCT that demonstrated DPC SSI of 12% versus 33% when primary closure is used with dirty wounds (p=0.01) [1]. Non-significance might however be due to the variability of sample size or contamination, which also confirmed no difference in appendicitis (12% DPC versus 18% PC; p=0.20) by Cohn *et al.* [3]. The increased SSIs in PC-D might be due to either drain-related infections or incomplete fluid evacuation, which is in line with a meta- analysis by De Simone *et al.* which found no SSI advantage of subcutaneous drains in abdominal surgery (RR=1.05, p=0.72) [16]. However, Fujii *et al.* also found the lower incidence of SSIs with drains in colorectal practice (4.5% versus 12.5%; p=0.03) [15], which implies effectiveness in selected procedures. Hospital stay was longer with DPC (12.4 versus 9.8 days) which is equivalent to the multicenter study (14 versus 10 days; p< 0.01) by Bhangu *et al.* [5] due to dressing changes and re-sutures, which raise costs in low-resource settings. Readmission equivalence (p=0.49) is in contrast to some reports of increased DPC readmission because of dehiscence [14]. The presence of covariates such as diabetes (OR=2.8) and obesity (OR=3.1) as independent variables confirms CDC [4] and cohort estimates (diabetes OR=2.2) made by Waisbren *et al.* [6]. The risk of Stoma (OR=2.6) represents the continued contamination [5], whereas low albumin is an indication of malnutrition that hinders healing [10]. The subgroup analysis that has supported DPC among diabetics (10% versus 30%; p=0.04) is consistent with the review by Ubbink *et al.* that recommends the use of DPC in high-risk populations [13]. A 2024 counterfactual prediction modelling study on wound closure in complicated appendicitis similarly demonstrated that patient-specific factors including diabetes, wound contamination class, and ruptured viscus independently modulated the benefit of DPC versus PC, reinforcing the need for individualized closure strategies [19]. There is no early SSI difference, which suggests the same initial contamination control, but additional long-term advantages to DPC. Our findings added to the literature on NPWT-aided DPC to minimize the occurrence of SSIs (5.6% versus 16.7%; p=0.02) [11], indicating that hybrid interventions be considered. A 2024 RCT from India specifically found that NPWT-assisted DPC reduced SSI to 10% versus 37.5% with conventional DPC alone (p=0.004) in grade IV abdominal wounds, along with significantly lower seroma and dehiscence rates [20]. Recent 2025 evidence further supports nuanced wound management: Mallaiah *et al.* showed that subcutaneous negative suction drainage significantly reduced SSI incidence (16.7% versus 26.7%, p=0.024), seroma formation, and wound dehiscence compared with no-drain closure in Indian emergency laparotomy for peritonitis [21]. Additionally, closed NPWT applied to the primarily sutured wound following emergency laparotomy for gastrointestinal perforation significantly reduced combined SSI rates (26.2% vs 45.2%, p=0.028) compared to standard dressing in a 2025 single-centre study [22], reinforcing the role of closed-suction and negative-pressure adjuncts in primary closure strategies for contaminated wounds. Furthermore, a 2025 case series confirmed the clinical and cost effectiveness of DPC for contaminated midline laparotomy incisions in perforated diverticulitis, noting avoidance of SSI in all three cases and reduced outpatient wound care burden [23]. Local comparative data, which are lacking globally according to systematic reviews, represent a novelty in India, where SSIs overwhelm healthcare (estimated 30-35% in emergencies) [8]. Evidence-based management algorithms for complicated intra-abdominal infections and contaminated wounds, such as those proposed by Sartelli *et al.* advocate for risk-stratified wound closure decisions and antibiotic stewardship that directly support the individualized approach demonstrated in the present study [24]

## Limitations: 

Single-center bias, no blinding, no microbiology.

## Strengths:

Multivariate analysis, randomization, standardized protocols. The controversy could be solved by future multicenter RCTs which include the VAC or antimicrobial-impregnated drains to achieve optimal results in contaminated wounds. Finally, customized approaches will be needed, such as the DPC-based on diabetics/obese, PC-D with faster discharge, which can be effective and efficient

## Conclusion:

DPC has a potential of reducing SSI compared to PC-D in contaminated laparotomies but this is not significant at the expense of increased stay. DPC is favoured in patients with high-risk factors such as diabetes. Individualized methods of closure are suggested and additional studies are needed.

## Advancement to knowledge:

This prospective randomized controlled trial provides contextual Indian tertiary care evidence on wound closure strategies in contaminated emergency laparotomies. It demonstrates that while DPC trends toward lower SSI rates compared to PC-D, the difference is not statistically significant and comes at the cost of prolonged hospitalization. Importantly, it identifies diabetes, obesity, and hypoalbuminemia as independent SSI predictors and shows a statistically significant benefit of DPC in the diabetic subgroup, supporting individualized, risk-stratified wound closure decision-making in resource-limited settings where such local data have hitherto been sparse.

## Declaration:

Authors contributed to design, execution, analysis and writing. Ethical compliance ensured.
